# Possible Serological Markers to Predict Mortality in Acute Exacerbation of Idiopathic Pulmonary Fibrosis

**DOI:** 10.3390/medicina55050132

**Published:** 2019-05-13

**Authors:** Yoshimasa Hachisu, Keisuke Murata, Kousuke Takei, Takuma Tsuchiya, Hiroaki Tsurumaki, Yasuhiko Koga, Takeo Horie, Atsushi Takise, Takeshi Hisada

**Affiliations:** 1 Department of Respiratory Medicine, Maebashi Red Cross Hospital, 389-1, Asakura-machi, Maebashi, Gunma 371-0811, Japan; keisuke.murata@maebashi.jrc.or.jp (K.M.); kousuke.takei@maebashi.jrc.or.jp (K.T.); t-tsuchiya@maebashi.jrc.or.jp (T.T.); t-horie@maebashi.jrc.or.jp (T.H.); at-takise@maebashi.jrc.or.jp (A.T.); 2 Department of Allergy and Respiratory Medicine, Gunma University Graduate School of Medicine, 3-39-15, Showa-machi, Maebashi, Gunma 371-8511, Japan; m12702056@gunma-u.ac.jp (H.T.); ykoga@gunma-u.ac.jp (Y.K.); 3 Gunma University Graduate School of Health Sciences, 3-39-22, Showa-machi, Maebashi, Gunma 371-8514, Japan; hisadat@gunma-u.ac.jp

**Keywords:** acute exacerbation, idiopathic pulmonary fibrosis, mortality, CRP, LDH, total cholesterol

## Abstract

*Background and objectives:* Idiopathic pulmonary fibrosis (IPF) has a particularly poor prognosis, and most IPF-related deaths are due to acute exacerbation (AE) of this condition. Few reports about biomarkers to predict prognosis of AE-IPF have been published since the release of the new AE-IPF criteria in 2016. The present study investigated relationships between serological markers and in-hospital mortality after the onset of AE-IPF. *Methods:* Demographic, serological, and imaging data from patients hospitalized at the Maebashi Red Cross Hospital (Gunma, Japan) between 1 January 2013, and 31 December 2017, were retrospectively reviewed. Subjects fulfilling the diagnostic criteria for AE-IPF were divided into those who survived or died; statistical analysis of risk factors was performed using data from these two groups. *Results:* Diagnostic criteria for AE-IPF were fulfilled by 84 patients (59 males (70.2%)), with a median age of 78 years (range, 56–95 years). IPF was diagnosed before hospitalization in 50 (59.5%) patients and 38 (45.2%) died in hospital. Among the serological markers at hospitalization in the deceased group, C-reactive protein (CRP) was significantly higher than in the survivor group (*p* = 0.002), while total serum protein (*p* = 0.031), albumin (*p* = 0.047) and total cholesterol (*p* = 0.039) were significantly lower. Cox hazard analysis of factors predicting mortality, corrected for age, sex and BMI, revealed the following: CRP (hazard ratio (HR) 1.080 (95% confidence interval (CI) 1.022–1.141); *p* = 0.006), LDH (HR 1.003 (95% CI 1.000–1.006); *p* = 0.037), and total cholesterol (HR 0.985 (95% CI 0.972–0.997); *p* = 0.018). *Conclusions:* Our data suggest that CRP, LDH, and total cholesterol may be biomarkers predicting mortality in patients with AE-IPF. However, only prospective controlled studies can confirm or not our observation as a generalizable one.

## 1. Introduction

Among the different types of idiopathic interstitial pneumonias, idiopathic pulmonary fibrosis (IPF) has an especially poor prognosis. Acute exacerbation (AE) is the cause of most IPF-associated deaths: the mortality rate among patients with IPF increases to 40–80% once AE has occurred [1,2,3]. Risk factors reported to be associated with death due to AE-IPF include low diffusing capacity of lungs for carbon monoxide, low forced vital capacity, smoking [4], elevated KL-6 levels, elevated high-resolution computed tomography score [5], low ratio of arterial partial pressure of oxygen to fraction of inspired oxygen (PaO_2_/FiO_2_) [6], and being over 60 years old [7]. There have been reports that pirfenidone administration before exacerbation [7], and recombinant thrombomodulin administration at the time of AE reduced the mortality rate [6]; however, no dramatic reduction in mortality has been achieved.

There have been numerous studies investigating the relationships between imaging and physiology tests and AE-IPF; however, reports describing the relationships between serological markers and AE-IPF have addressed only KL-6 and a small number of other markers. In addition, the number of published reports relating to the most recent AE-IPF diagnostic criteria, released in 2016 [8], is limited.

The present study was a retrospective investigation of the relationships between serological/physiological markers and the onset of AE-IPF defined by the most recent diagnostic criteria [8], and an evaluation of factors related to mortality.

## 2. Materials and Methods

### 2.1. Subjects

The present investigation was a single-center, retrospective study. Patients who were hospitalized at Maebashi Red Cross Hospital (Gunma Prefecture, Japan) between 1 January 2013, and 31 December 2017, were evaluated; those who fulfilled the diagnostic criteria for AE-IPF were included in the study. All participants in this study were confirmed to have met the 2016 AE-IPF diagnostic criteria at the enrollment of this study. We diagnosed IPF with a multidisciplinary approach. At least two respiratory specialists and a radiology specialist discussed and reviewed subjects. A histologist was also consulted when histological data were obtained.

The AE-IPF diagnostic criteria, as described by Collard et al. in 2016 [8], were indicators of acute and clinically significant deterioration in respiratory condition that characterizes new extensive alveolar opacity, with subjects required to fulfill the following criteria: IPF was diagnosed at the time of AE or had been diagnosed previously; acute progression of dyspnea had occurred within the previous month; high-resolution computed tomography revealed signs to be consistent with the usual interstitial pneumonia pattern, in addition to new ground-glass and/or infiltrative opacities; deterioration that could not be explained solely on the basis of heart failure or excess fluid had accumulated. The usual treatment methods for AE-IPF were pulse intravenous methylprednisolone and high-dose corticosteroid [9,10]. All subjects in the present study were treated by either method after diagnosis.

This study was reviewed and approved by the Ethics Committee of Maebashi Red Cross Hospital on 12 July 2018 (acceptance no. 30-19).

### 2.2. Clinical Assessment

The following data were obtained from medical records: demographic information (age at hospitalization, sex, height, body weight); sociological information (medical history, smoking status, alcohol consumption status); IPF treatment before admission; treatment in hospital; serological test results in hospitalization; arterial blood gas analysis results; imaging results; and treatment progression. Body mass index (BMI) and body surface area were calculated from data. PaO_2_/FiO_2_ and alveolar-arterial oxygen difference (AaDO_2_) were calculated from arterial blood gas analysis results. In addition, the duration of hospitalization, and whether death occurred while in hospital, were obtained from the medical records.

### 2.3. Statistical Analysis

Statistical analysis was performed using R version 3.4.1 and EZR version 1.37. Continuous variables were analyzed and compared using the Mann–Whitney-U test and expressed as median (maximum and minimum). Nominal variables were analyzed and compared using the Fisher’s exact test and expressed as numbers and ratios; *p* < 0.05 was considered to be statistically significant.

Cox hazard analysis was performed, and corresponding hazard ratio (HR) and 95% confidence interval (CI) were calculated. For parameters that demonstrated significant differences, factors previously demonstrated to be correlated with AE-IPF mortality were included. Receiver operating characteristic analysis was performed using continuous variables that were judged to be significant factors, and cut-off values were calculated.

## 3. Results

Of the patients who were hospitalized at the Maebashi Red Cross Hospital between 1 January 2013, and 31 December 2017, consecutive 84 patients met the diagnostic criteria for AE-IPF and were included in the present study (Table 1). The median age was 78 years (range, 56–95 years), 59 (70.2%) subjects were male, and 50 (59.5%) were diagnosed with IPF before hospitalization. Of the subjects who were diagnosed with IPF before hospitalization, 4 (8%) were administered antifibrotic agents and 13 (26%) were administered systemic corticosteroids, whereas 33 (66%) were not treated for IPF. The median duration of hospitalization was 25 days (range, 2–113 days), and 38 (45.2%) subjects died in hospital. Extracorporeal membrane oxygenation was not performed in any subjects.

The subjects were divided into two groups: those who died (deceased); and those who survived (survivor). Statistical analysis was performed using data from these groups. As shown in Table 2, there were no significant differences between the groups in sex, age, or BMI, or in whether the subjects smoked or consumed alcohol. The proportion of subjects in whom home oxygen therapy was prescribed before AE-IPF tended to be lower in the deceased group (5 (13.2%) versus 14 (30.4%), respectively); however, the difference was not statistically significant (*p* = 0.071). No differences in the history of diabetes, dyslipidemia, myocardial infarction, cerebral infarction, malignant tumors, or bronchial asthma were found between the deceased and survivor groups. Regarding serological markers at hospitalization, c-reactive protein (CRP, mg/dL) was significantly higher in the deceased group (11.57 (95% CI 0.77 to 27.73) versus 6.69 (95% CI 0.1–21.84); *p* = 0.002), and serum total protein (TP, g/dL), albumin (Alb, g/dL), and total cholesterol (T-chol, mg/dL) were significantly lower (6.5 (5.2–8.4) versus 6.8 (5.5–9.4); *p* = 0.031), 2.75 (1.7–3.5) versus 2.95 (2–4.1); *p* = 0.047), and 133 (82–477) versus 159 (66–222); *p* = 0.039), respectively. Surfactant protein-A (SP-A, ng/mL) tended to be higher in the deceased group (124.2 (30.4–251) versus 91.2 (27–270.7)); however, the difference was not significant (*p* = 0.067). No significant differences in KL-6 or surfactant protein D (SP-D) were found. Arterial blood gas analysis revealed no significant differences in PaO_2_/FiO_2_ ratio or AaDO_2_.

No significant differences between the groups were found in the number of subjects who were admitted to the intensive care unit, or in whom tracheal intubation or nasal high-flow therapy were performed. The number of subjects in whom non-invasive positive-pressure ventilation was performed tended to be higher in the deceased group (8 (21.1%) versus 3 (6.5%)); however, the difference was not significant (*p* = 0.059). No significant differences were found in the number of subjects who underwent steroid pulse therapy, cyclophosphamide pulse therapy, or recombinant thrombomodulin administration. The number of subjects administered sivelestat was significantly higher in the deceased group (7 (18.4%) versus 1 (2.2%); *p* = 0.02). We confirmed the presence or absence of malignant tumor from medical records. Fifteen and nine patients had malignancies as comorbidities and past tumors, respectively. The most frequent comorbid/past tumor was lung cancer, which was observed in 11 patients.

Along with data obtained from the pulmonary function test (Table 3), no significant differences were found between the deceased and survivor groups of patients who were diagnosed with AE-IPF before hospitalization.

**Figure 1:**
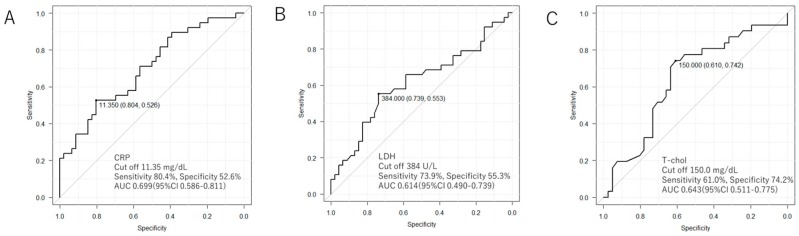
Receiver operating characteristic: ROC analysis about prognostic factors. These figures reveal about (**A**) CRP, (**B**) LDH, and (**C**) total cholesterol. For all item cut off values are calculated from the Youden index.

Serological markers that were shown in the Mann–Whitney test to be *p* < 0.05 (CRP, Alb, and T-chol), and KL-6 and lactate dehydrogenase (LDH), which were already known to be correlated with mortality, were corrected for age, sex, and BMI, and Cox hazard analysis was performed (Table 4). From the point of the collinearity of TP and Alb, TP was excluded. The results revealed that CRP (HR 1.080 (95% CI 1.022–1.141); *p* = 0.006), LDH (HR 1.003 (95% CI 1.000–1.006); *p* = 0.037), and T-chol (HR 0.985 (95% CI 0.972–0.997); *p* = 0.018) were predictive factors for time until death. Receiver operating characteristic analysis was performed, and the cut-off values of the mortality-predicting continuous variables were calculated (Figure 1) as follows: CRP: 11.35 mg/dL (sensitivity, 80.4%; specificity, 52.6%); LDH: 384 U/L (sensitivity: 73.9%; specificity: 55.3%); and T-chol: 150 mg/dL (sensitivity: 61.0%; specificity: 74.2%).

All patients were divided into four groups based on the obtained CRP and cholesterol cutoff values and compared for death. As shown in Table 5, the group with low total cholesterol and high CRP had a poorer prognosis relative to that of the group with high cholesterol and low CRP. 

## 4. Discussion

AE-IPF accounts for 15% to 40% of IPF-related deaths [11,12]. In the past, a patient was diagnosed with AE-IPF when the following criteria were met [13]: diagnosis of IPF has been made; dyspnea has worsened within the previous 30 days; gas exchange function has depressed, in that the PaO_2_/FiO_2_ ratio and/or arterial partial pressure of oxygen (PaO_2_) has been low; new alveolar lesions have developed; and other causative diseases, such as infection and heart failure, could be ruled out.

Single-center studies by Kataoka et al. and Sakamoto et al. reported 44 cases and 80 cases of AE-IPF in five years and 10 years, respectively [6,14]. Thus, by comparison to such previous reports, the number of patients in our study is indeed high. Our hospital is a tertiary care hospital with an advanced critical care center and accepts over 6000 ambulances a year. Therefore, it also received more AE-IPF patients than do other hospitals. Moreover, this study followed the AE-IPF criteria in 2016 and included patients with triggered AE. Many patients were also diagnosed with IPF at the time of hospitalization because patients who had never been diagnosed with or treated for IPF had often visited the emergency department prior to hospitalization. Hence, 40% of the whole population having been diagnosed at the time of hospitalization may be partly accounted for by the characteristics of our hospital.

The new diagnostic criteria proposed in 2016 widened the range of AE-IPF [8], in that AE-IPF triggered by infection, surgery or drugs were included, regardless of whether IPF had been diagnosed previously. It has been reported that this triggered AE tends to involve more extensive opacity, and a shorter life expectancy than untriggered AE [15], and it is possible that there are differences in progression between different types of AE-IPF. 

In addition, among the patients who had already been diagnosed, only four patients used antifibrotic drugs, and many patients remained untreated. It is plausible that primary care physicians did not introduce antifibrotic drugs into the treatment of elderly patients in consideration of the drug’s side effects and that non-specialists administered corticosteroids according to past recommendations, despite not corticosteroids but antifibrotic drugs having been recommended for IPF treatment. Moreover, while anti-fibrotic drugs suppress the incidence of AE, the corticosteroid administration group exhibited an increase in the incidence of AE; this may account for the limited prescription of antifibrotic drugs in this study.

Previously, low forced vital capacity [1], elevated AaDO2 [16], elevated KL-6 levels, and pulmonary hypertension [17] have been proposed as risk factors for the onset of AE-IPF. The antifibrotic agent, nintedanib has been reported to exert suppressive effects on AE-IPF [18] and is expected to regulate IPF pathology. In addition, it has also been reported that pirfenidone administration reduced mortality after AE-IPF [19], and improvements in prognosis are expected, although there was no inhibitory effect on AE-IPF onset by pirfenidone in a randomized study [20].

The relationships between serological markers and AE-IPF were investigated in this study, and findings suggested that CRP, LDH, and T-chol might be predictive factors for in-hospital mortality after AE-IPF. KL-6, LDH, and CRP have been known to be mortality-predicting factors at AE-IPF onset [2,3,14].

CRP is a protein that is produced by hepatocytes in response to stimulation by cytokines such as tumor necrosis factor-alpha, interleukin-1, and interleukin-6. During the chronic progression of interstitial pneumonia, it has been reported that a baseline CRP level associated with scleroderma was a long-term prognostic factor [21], and that pirfenidone administration for IPF reduced the levels of inflammatory markers including CRP [22], suggesting that there may be a relationship between the progression of interstitial pneumonia and serum CRP level. In the case of AE-IPF, there have been several reports that high serum CRP level was a prognostic factor, and Song et al. proposed serum CRP and bronchoalveolar lavage lymphocyte count as predictive factors for in-hospital death due to AE-IPF [1]. Furthermore, Kataoka et al. reported that administration of recombinant thrombomodulin and serum CRP level were correlated with the survival rate for 3 months after the onset of AE-IPF [14]. Findings from the present study support these previous studies, and it is considered that, even in relation to the new AE-IPF diagnostic criteria, CRP may be effective for AE-IPF prognosis and prediction.

It has been reported that elevation of serum LDH level in interstitial lung disease was a predictive factor for the onset of AE [23] and elevation of LDH in scleroderma lung might be a marker of pulmonary fibrosis [24]. In AE-IPF, LDH has been reported to be a predictive factor for survival [3], and the association between interstitial pneumonia and LDH has been clearly demonstrated.

There have been virtually no reports addressing the relationship between serum cholesterol level and AE-IPF. Correlations between low serum cholesterol level and mortality rate are known in several diseases, and it has been reported that hypocholesterolemia was an independent mortality-predicting factor with malignant tumors [25] and that low-density lipoprotein cholesterol and T-chol were mortality-predicting factors in patients undergoing dialysis [26]. If hypocholesterolemia progresses in systemic diseases due to chronic exhaustion and is associated with mortality, a similar correlation between hypocholesterolemia progression and mortality can be explained in IPF patients. The present study demonstrated that T-chol at the time of AE-IPF onset has a relationship with a predictive factor for in-hospital mortality. The acute or chronic decrease in serum T-chol may be a prognostic marker for predicting the onset or the mortality of AE-IPF.

It may be difficult to predict the prognosis of AE-IPF by using a single biomarker because various causes contribute to the development of AE-IPF. It has previously been reported that hypolipidemia was a risk factor for respiratory tract infection; it impairs immune responses to lung infection, resulting in the development of respiratory tract infection [27]. Low total cholesterol is also reported to be a biomarker predictive of poor prognoses in patients with lower respiratory tract infections [28]. In this study, the median CRP, LDH, and total cholesterol in the deceased group were 11.57 mg/dL, 386 U/L, and 133 mg/dL, respectively (Table 2). These median values are similar to the cut-off value (Figure 1). The combination of these cut-off values may thus be useful to predict the prognoses in a single patient with AE-IPF. As shown in Table 5, the group with low total cholesterol and high CRP had a poorer prognosis relative to that of the group with high cholesterol and low CRP; the former may reflect a poor prognosis due to severe infection-triggered AE-IPF rather than untriggered AE-IPF. 

Consistent with this supposition, experimental and clinical investigations have revealed that high levels of cytokines, such as CRP, decreased circulating cholesterol levels during severe infection [29]. Sepsis also decreases serum concentrations of total cholesterol and albumin [30]. Furthermore, multivariate analysis revealed that low total cholesterol (of less than 150 U/mL) featured poor prognostic value in AE-COPD patients [31]. Moreover, Chien et al. reported that non-survivors due to severe community-acquired pneumonia exhibited higher CRP level and lower serum HDL and LDL cholesterol levels [32]. Therefore, intensive, continuous antibiotic treatment in tandem with steroid-pulse therapy may help to promote a better prognosis of AE-IPF in the group with low total cholesterol and high CRP. Further prospective studies are required to clarify the significance of these biomarkers to the prediction of the prognosis of AE-IPF.

In addition to the presence of untriggered AE, this study included triggered AE caused by various factors, including infection and operation. Due to the heterogeneity among the pathologies of patients with AE-IPF, a single biomarker cannot reflect the various factors underlying the development of AE-IPF; hence, the AUC and specificity were relatively low.

There are a few limitations in this study. Because this is a small, single-center retrospective study, the study population may not represent the entire AE-IPF patient population. In particular, a few patients in this study were treated with antifibrotic drugs. The administration of antifibrotic drugs is currently standard for the treatment of IPF; future analyses should thus be performed with populations featuring a greater proportion of patients treated with antifibrotic drugs. In addition, we were able to analyze serological markers only at admission and could not, therefore, record continuous changes in the levels of serological markers. This study cannot confirm serial changes of serological markers during AE. Therefore, the results in this study should be confirmed in a large size and in a multi-center study. In this study, we obtained spirometry data of only part of IPF patients before the onset of AE-IPF. Many of the 50 cases diagnosed with IPF before hospitalization were followed up at the local clinic when the IPF was at a stable condition and hence we could not obtain the data regarding spirometry performed at the clinic; alternatively in some other cases, a follow-up spirometry was not adequate at that time due to AE-IPF onset. Follow-up of spirometry in IPF patients is crucial as low forced vital capacity is associated with the onset of AE-IPF [1], and low diffusing capacity of lungs for carbon monoxide is also associated with death on AE-IPF [4]. Furthermore, it remains unclear whether infection-triggered AE-IPF with high serum CRP levels has a poor prognosis; since, in most of our cases, the presence of an infectious disease was not confirmed using serum procalcitonin levels or bacterial culture tests using bronchoalveolar lavage fluid. Therefore, further studies designed to detect infection-triggered AE-IPF and including follow-up of spirometry tests are required to confirm our results and to discover better biomarkers of AE-IPF. However, elevated CRP and LDH levels were consistent with previous reports describing a prognostic biomarker of AE-IPF defined prior to the definition by Collard et al. in 2016 [8]. Therefore, further prospectively designed studies should be required to confirm our results and may promote more confident predictive biomarkers of AE-IPF.

## 5. Conclusions

Our data suggest that CRP, LDH, and total cholesterol may be biomarkers predicting mortality in patients with AE-IPF. However, only prospective controlled studies can confirm or not our observation as a generalizable one.

## Figures and Tables

**Table 1:** Background and treatment progression of all patients.

Background	*n* = 84
Age	78 (56, 95)
Male	59 (70.2)
BMI (kg/m^2^)	20.95 (13.06, 33.64)
BSA (m^2^)	1.53 (1.1, 2.09)
Diagnosed IPF before AE	50 (59.5)
Treatment to IPF	17 (34)
Anti-fibrotic agent	4 (8)
Corticosteroid	13 (26)
Not treatment	33 (66)
Smoking	65 (77.4)
Alcohol	30 (35.7)
Home oxygen therapy	19 (22.6)
**Medical history**	
Diabetes mellitus	23 (27.4)
Dyslipidemia	15 (17.9)
Cerebral infarction	11 (13.1)
Myocardial infarction	4 (4.8)
Bronchial asthma	4 (4.8)
Malignant tumor	24 (28.6)
**Serological marker**	
WBC (/μL)	10,900 (3500, 31,000)
Total protein (g/dL)	6.65 (5.2, 9.4)
Albumin (g/dL)	2.9 (1.7, 4.1)
LDH (U/L)	337.5 (150, 906)
Calcium (mg/dL)	9.4 (8.3, 10.3)
CRP (mg/dL)	8.47 (0.1, 27.73)
D-dimer (μg/mL)	4.4 (0.8, 49)
Blood sugar (mg/dL)	131 (79, 477)
Hemoglobin A1c (%)	5.8 (4.8, 9.9)
Total cholesterol (mg/dL)	146.5 (66, 248)
KL-6 (U/mL)	1032.5 (165, 5362)
SP-A (ng/mL)	100.8 (27, 270.7)
SP-D (ng/mL)	363 (118, 1760)
**BGA in artery**	
pH	7.46 (7.03, 7.56)
PaCO_2_ (mmHg)	36.2 (17.1, 65.5)
PaO_2_ (mmHg)	64.5 (32.2, 188.1)
Base excess (mmol/L)	1.8 (−15.50, 12.6)
Lactate (mmol/L)	1.63 (0.51, 9.67)
AaDO_2_	130.34 (15.88, 621.42)
PaO_2_/FiO_2_	186.00 (45.15, 406.67)
**Treatment in hospital**	
Treatment in ICU	3 (3.6)
Mechanical ventilation	4 (4.8)
NPPV	11 (13.1)
Nasal high flow	28 (33.3)
ECMO	0 (0)
Steroid pulse therapy	50 (59.5)
Cyclophosphamide pulse therapy	10 (11.9)
Sivelestat	8 (9.5)
Recombinant thrombomodulin	3 (3.6)
Anti-fibrotic agent	4 (4.8)
Length of hospital day	25 (2, 113)
Death during hospitalization	38 (45.2)

Serological marker and blood gas analysis were first data in hospitalization. Values are median (minimum, maximum) or number (percentage). BMI: body mass index, BSA: body surface area, IPF: idiopathic pulmonary fibrosis, AE: acute exacerbation, WBC: white blood cell, LDH: lactate dehydrogenase, CRP: C-reactive protein, KL-6: Krebs von den lungen-6, SP-A: surfactant protein-A, SP-D: surfactant protein-D, BGA: blood gas analysis, AaDO2: alveolar-arterial oxygen difference, ICU: intensive care unit, NPPV: non-invasive positive pressure ventilation, ECMO: extracorporeal membrane oxygenation.

**Table 2:** Comparison between survival group and death group in acute exacerbation- Idiopathic pulmonary fibrosis (AE-IPF).

Factor	Survival (*n* = 46)	Death (*n* = 38)	*p* Value
**Background factor**			
Age	80 (61, 91)	77 (56, 95)	0.316
Male	31 (67.4)	28 (73.7)	0.634
BMI (kg/m^2^)	20.45 (13.06, 33.64)	21.94 (13.25, 30.87)	0.128
BSA (m^2^)	1.52 (1.1, 1.94)	1.55 (1.22, 2.09)	0.267
Diagnosis before AE	30 (65.2)	20 (52.6)	0.271
Smoking	35 (76.1)	30 (78.9)	0.799
Alcohol	18 (39.1)	12 (31.6)	0.502
Home oxygen therapy	14 (30.4)	5 (13.2)	0.071
**Medical history**			
Diabetes mellitus	14 (30.4)	9 (23.7)	0.624
Dyslipidemia	8 (17.4)	7 (18.4)	1
Myocardial infarction	2 (4.3)	2 (5.3)	1
Cerebral infarction	6 (13)	5 (13.2)	1
Bronchial asthma	3 (6.5)	1 (2.6)	0.623
Malignant tumor	15 (32.6)	9 (23.7)	0.468
**Treatment in hospital**			
Treatment in ICU	1 (2.2)	2 (5.3)	0.587
Mechanical ventilation	1 (2.2)	3 (7.9)	0.324
NPPV	3 (6.5)	8 (21.1)	0.059
Nasal high flow	12 (26.1)	16 (42.1)	0.164
Steroid pulse therapy	24 (52.2)	26 (68.4)	0.181
Cyclophosphamide pulse therapy	3 (6.5)	7 (18.4)	0.174
Sivelestat	1 (2.2)	7 (18.4)	0.02 *
Recombinant thrombomodulin	2 (4.3)	1 (2.6)	1
Anti-fibrotic agent	2 (4.3)	2 (5.3)	1
**Serological marker**			
WBC(/μL)	10,300 (5800, 22,700)	11,150 (3500, 31,000)	0.406
Total protein (g/dL)	6.80 (5.5, 9.4)	6.50 (5.2, 8.4)	0.031 *
Albumin (g/dL)	2.95 (2, 4.1)	2.75 (1.7, 3.5)	0.047 *
LDH (U/L)	315.5 (150, 619)	386.5 (157, 906)	0.073
Calcium (mg/dL)	9.3 (8.3, 10)	9.50 (8.7, 10.3)	0.429
CRP (mg/dL)	6.69 (0.1, 21.84)	1.57 (0.77, 27.73)	0.002 **
D-dimer (μg/mL)	3.3 (0.8, 34.2)	5.35 (0.9, 49)	0.152
Blood sugar (mg/dL)	133 (79, 330)	130.0 (82, 477)	0.728
Hemoglobin A1c (%)	5.9 (4.8, 7.8)	5.80 (5.4, 9.9)	0.848
Total cholesterol (mg/dL)	159 (66, 222)	133.0 (88, 248)	0.039 *
KL-6 (U/mL)	1027 (248, 3936)	1085.0 (165, 5362)	0.448
SP-A (ng/mL)	91.2 (27, 270.7)	124.2 (30.4, 251)	0.067
SP-D (ng/mL)	354 (128, 1760)	378.0 (118, 1060)	0.872
**BGA in artery**			
pH	7.45 (7.23, 7.56)	7.46 (7.03, 7.54)	0.389
PaCO_2_ (mmHg)	36.4 (23.2, 57.8)	35.3 (17.1, 65.5)	0.909
PaO_2_ (mmHg)	61.3 (32.2, 188.1)	6.25 (34.2, 91)	0.983
Base excess (mmol/L)	1.8 (−7.1, 12.6)	1.75 (−15.5, 10.8)	0.628
Lactate (mmol/L)	1.74 (0.83, 7.15)	1.6 (0.51, 9.67)	0.754
AaDO2	117.88 (16.2, 621.42)	207.47 (15.88, 617.85)	0.229
PaO_2_/FiO_2_	191.25 (45.15, 406.67)	146.58 (45.25, 377.14)	0.17

Values are median (minimum, maximum) or number (percentage). The nominal variables are analyzed Fisher’s exact test and the continuous variables are analyzed by Mann–Whitney-U test. * *p* < 0.05, ** *p* < 0.01.

**Table 3:** Lung function test within 6 months before AE-IPF onset.

Factor	Survival (*n* = 14)	Death (*n* = 6)	*p* Value
Pulmonary function test			
VC(L)	1.99 (1.21, 3.94)	2.74 (1.67, 3.17)	0.232
FVC(L)	2.07 (1.26, 3.79)	2.40 (1.85, 3.14)	0.481
FEV1.0(L)	1.76 (1.20, 2.65)	1.86 (1.26, 2.78)	0.244
DLCO	8.82 (4.03, 9.72)	8.32 (7.81, 8.82)	0.769

VC(L) = vital capacity; FVC(L) = forced vital capacity; FEV1.0(L) = forced expiratory volume in one second; DLCO = diffusing capacity of the lungs for carbon monoxide.

**Table 4:** Cox regression analysis about prognostic factors for in-hospital mortality of patients with acute exacerbation.

	HR (95% CI)	*p* Value
CRP	1.080 (1.022–1.141)	0.006 **
LDH	1.003 (1.000–1.006)	0.037 *
T-chol	0.985 (0.972–0.997)	0.018 *

* p < 0.05, ** *p* < 0.01 CRP: c-reactive protein, LDH: lactate dehydrogenase, T-chol: total cholesterol. Wald’s test *p* = 0.003.

**Table 5:** The number of deceased cases separated from cut-off values of total cholesterol and c-reactive protein (CRP).

	CRP ≥ 11.35 mg/dL	CRP < 11.35 mg/dL
T-chol <150 mg/dL	17/22 (77.3%)	6/17 (35.3%)
T-chol ≥150 mg/dL	1/5 (20.0%)	7/28 (25.0%)

## References

[1] Song J.W., Hong S.B., Lim C.M., Koh Y., Kim D.S. (2011). Acute exacerbation of idiopathic pulmonary fibrosis: incidence, risk factors and outcome. Eur. Respir. J..

[2] Kishaba T., Tamaki H., Shimaoka Y., Fukuyama H., Yamashiro S. (2014). Staging of acute exacerbation in patients with idiopathic pulmonary fibrosis. Lung.

[3] Isshiki T., Sakamoto S., Kinoshita A., Sugino K., Kurosaki A., Homma S. (2015). Recombinant Human Soluble Thrombomodulin Treatment for Acute Exacerbation of Idiopathic Pulmonary Fibrosis: A Retrospective Study. Respiration.

[4] Bi Y., Rekić D., Wang Y., Karimi-Shah B.A., Chowdhury B.A., O Paterniti M. (2017). Acute Exacerbation and Decline in Forced Vital Capacity Are Associated with Increased Mortality in Idiopathic Pulmonary Fibrosis. Ann. Am. Thorac. Soc..

[5] Fujimoto K., Taniguchi H., Johkoh T., Kondoh Y., Ichikado K., Sumikawa H., Ogura T., Kataoka K., Endo T., Kawaguchi A. (2012). Acute exacerbation of idiopathic pulmonary fibrosis: high-resolution CT scores predict mortality. Eur. Radiol..

[6] Sakamoto S., Shimizu H., Isshiki T., Sugino K., Kurosaki A., Homma S. (2018). Recombinant human soluble thrombomodulin for acute exacerbation of idiopathic pulmonary fibrosis: A historically controlled study. Respir. Investig..

[7] Zubairi A.B.S., Ahmad H., Hassan M., Sarwar S., Abbas A., Shahzad T., Irfan M. (2018). Clinical characteristics and factors associated with mortality in idiopathic pulmonary fibrosis: An experience from a tertiary care center in Pakistan. Clin. Respir. J..

[8] Collard H.R., Ryerson C.J., Corte T.J., Jenkins G., Kondoh Y., Lederer D.J., Lee J.S., Maher T.M., Wells A.U., Antoniou K.M. (2016). Acute Exacerbation of Idiopathic Pulmonary Fibrosis. An International Working Group Report. Am. J. Respir. Crit. Care Med..

[9] Agarwal R., Jindal S.K. (2008). Acute exacerbation of idiopathic pulmonary fibrosis: A systematic review. Eur. J. Intern. Med..

[10] Nishiyama O., Shimizu M., Ito Y., Kume H., Suzuki R., Yokoi T., Yamaki K. (2001). Effect of prolonged low-dose methylprednisolone therapy in acute exacerbation of idiopathic pulmonary fibrosis. Respir. Care.

[11] Natsuizaka M., Chiba H., Kuronuma K., Otsuka M., Kudo K., Mori M., Bando M., Sugiyama Y., Takahashi H. (2014). Epidemiologic Survey of Japanese Patients with Idiopathic Pulmonary Fibrosis and Investigation of Ethnic Differences. Am. J. Respir. Crit. Care Med..

[12] Fernandez Perez E.R., Daniels C.E., Schroeder D.R., St Sauver J., Hartman T.E., Bartholmai B.J., Yi E.S., Ryu J.H. (2010). Incidence, prevalence, and clinical course of idiopathic pulmonary fibrosis: a population-based study. Chest.

[13] Collard H.R., Moore B.B., Flaherty K.R., Brown K.K., Kaner R.J., King T.E., Lasky J.A., Loyd J.E., Noth I., Olman M.A. (2007). Acute Exacerbations of Idiopathic Pulmonary Fibrosis. Am. J. Respir. Crit. Care Med..

[14] Kataoka K., Taniguchi H., Kondoh Y., Nishiyama O., Kimura T., Matsuda T., Yokoyama T., Sakamoto K., Ando M. (2015). Recombinant Human Thrombomodulin in Acute Exacerbation of Idiopathic Pulmonary Fibrosis. Chest.

[15] Kishaba T., Nei Y., Momose M., Nagano H., Yamashiro S. (2018). Clinical Characteristics Based on the New Criteria of Acute Exacerbation in Patients with Idiopathic Pulmonary Fibrosis. Eurasian J. Med..

[16] Kondoh Y., Taniguchi H., Ebina M., Azuma A., Ogura T., Taguchi Y., Suga M., Takahashi H., Nakata K., Sugiyama Y. (2015). Risk factors for acute exacerbation of idiopathic pulmonary fibrosis – Extended analysis of pirfenidone trial in Japan. Respir. Investig..

[17] Qiu M., Chen Y., Ye Q. (2018). Risk factors for acute exacerbation of idiopathic pulmonary fibrosis: A systematic review and meta-analysis. Clin. Respir. J..

[18] Richeldi L., Du Bois R.M., Raghu G., Azuma A., Brown K.K., Costabel U., Cottin V., Flaherty K.R., Hansell D.M., Inoue Y. (2014). Efficacy and Safety of Nintedanib in Idiopathic Pulmonary Fibrosis. New Engl. J. Med..

[19] Furuya K., Sakamoto S., Shimizu H., Sekiya M., Kinoshita A., Isshiki T., Sugino K., Matsumoto K., Homma S. (2017). Pirfenidone for acute exacerbation of idiopathic pulmonary fibrosis: A retrospective study. Respir. Med..

[20] Taniguchi H., Ebina M., Kondoh Y., Ogura T., Azuma A., Suga M., Taguchi Y., Takahashi H., Nakata K., Sato A. (2010). Pirfenidone in idiopathic pulmonary fibrosis. Eur. Respir. J..

[21] Liu X., Mayes M.D., Pedroza C., Draeger H.T., Gonzalez E.B., Harper B.E., Reveille J.D., Assassi S. (2013). Does C-Reactive Protein Predict the Long-Term Progression of Interstitial Lung Disease and Survival in Patients With Early Systemic Sclerosis?. Arthritis Care Res..

[22] Matsumura T., Tsushima K., Abe M., Suzuki K., Yamagishi K., Matsumura A., Ichimura Y., Ikari J., Terada J., Tatsumi K. (2018). The effects of pirfenidone in patients with an acute exacerbation of interstitial pneumonia. Clin. Respir. J..

[23] Ishikawa G., Acquah S.O., Salvatore M., Padilla M.L. (2017). Elevated serum D-dimer level is associated with an increased risk of acute exacerbation in interstitial lung disease. Respir. Med..

[24] Tanaseanu C.M., Tiglea I.A., Marta D.S., Dumitrascu A.L., Popescu M., Tanaseanu S., Moldoveanu E. (2015). Lactate dehydrogenase a possible marker of progressive microvasculopathy and interstitial lung disease in systemic sclerosis. Acta Med. Mediterr..

[25] Palmier J., Lanzrath B.J. (2012). Laboratory and biometric predictors of cancer-related mortality in an insured population. J. Insur. Med..

[26] Chiang C.-K., Ho T.-I., Hsu S.-P., Peng Y.-S., Pai M.-F., Yang S.-Y., Hung K.-Y., Tsai T.-J. (2005). Low-Density Lipoprotein Cholesterol: Association with Mortality and Hospitalization in Hemodialysis Patients. Blood Purif..

[27] Birrell M.A., Catley M.C., Hardaker E., Wong S., Willson T.M., McCluskie K., Leonard T., Farrow S.N., Collins J.L., Haj-Yahia S. (2007). Novel Role for the Liver X Nuclear Receptor in the Suppression of Lung Inflammatory Responses. J. Boil. Chem..

[28] Gruber M., Christ-Crain M., Stolz D., Keller U., Müller C., Bingisser R., Tamm M., Mueller B., Schuetz P. (2009). Prognostic impact of plasma lipids in patients with lower respiratory tract infections—An observational study. Swiss Med. Wkly..

[29] Alvarez C., Ramos A. (1986). Lipids, lipoproteins, and apoproteins in serum during infection. Clin. Chem..

[30] Mooser V., Berger M.M., Tappy L., Cayeux C., Marcovina S.M., Darioli R., Nicod P., Chioléro R. (2000). Major Reduction in Plasma Lp(a) Levels During Sepsis and Burns. Arter. Thromb. Vasc. Boil..

[31] Fruchter O., Yigla M., Kramer M.R. (2015). Lipid Profile and Statin Use: The Paradox of Survival After Acute Exacerbation of Chronic Obstructive Pulmonary Disease. Am. J. Med Sci..

[32] Chien Y.-F., Chen C.-Y., Hsu C.-L., Chen K.-Y., Yu C.-J. (2015). Decreased serum level of lipoprotein cholesterol is a poor prognostic factor for patients with severe community-acquired pneumonia that required intensive care unit admission. J. Crit. Care.

